# Comparative evaluation of compressive and flexural strength, fluoride release and bacterial adhesion of GIC modified with CPP-ACP, bioactive glass, chitosan and MDPB

**DOI:** 10.34172/joddd.2021.004

**Published:** 2021-02-13

**Authors:** Natarajan Kirthika, Sampath Vidhya, Venkatappan Sujatha, Sekar Mahalaxmi, Renganathan Senthil Kumar

**Affiliations:** ^1^Department of Conservative Dentistry and Endodontics, Karpaga Vinayaga Institute of Dental Sciences, Tamil Nadu, India; ^2^Department of Conservative Dentistry and Endodontics, SRM Dental College, SRM Institute of Science and Technology, Chennai, India; ^3^Department of Conservative Dentistry and Endodontics, Adhiparasakthi Dental College & Hospital, Melmaruvathur, India

**Keywords:** Bacterial adhesion, Bioactive glass, Chitosan, CPP-ACP, Fluoride release, Glass-ionomer cement, MDPB

## Abstract

**Background.** This study evaluated the incorporation of casein phosphopeptide-amorphous calcium phosphate (CPP-ACP), calcium sodium phosphosilicate bioactive glass (BAG), chitosan (CH), and methacryloyloxydodecylpyridinium bromide (MDPB) on the compressive and flexural strength, fluoride (F^‒^ ) release, and bacterial adhesion of conventional glass-ionomer cement (C-GIC).

**Methods.** Modifications were implemented by adding CPP-ACP, BAG, and CH to the glass powder, while MDPB-GIC was prepared by incorporating MDPB to the liquid of C-GIC. Custom-made molds were used for specimen preparation. Compressive and flexural strengths were evaluated using a universal testing machine. F^‒^ release was calculated with Erichrome cyanide reagent, using UV-spectrophotometry, at two time intervals of 24 hours and seven days. For bacterial adhesion, the test specimens were exposed to the bacterial suspension of *Streptococcus mutans* and *Lactobacillus acidophilus* for 4 hours, and the adherent bacteria were quantified using colorimetry as the optical density (OD).

**Results.** The incorporation of MDPB increased the flexural strength of C-GIC, with no effect on its compressive strength. CH significantly improved the compressive and flexural strength; modifications with CPP-ACP, BAG, and MDPB significantly improved the flexural strength of C-GIC. While MDPB-GIC released significantly higher F^‒^ at 24 hours, CPP-ACP- and BAG-modified GICs were comparable to C-GIC on day 7. C-GIC exhibited the highest bacterial adhesion, and MDPB-GIC showed the least. The data were analyzed with one-way (ANOVA), and pairwise comparisons were made with Tukey HSD tests.

**Conclusion.** Hence, it can be concluded that the incorporation of CPP-ACP, BAG, and CH improved the mechanical properties of C-GIC, whereas MDPB improved the resistance of C-GIC to bacterial adhesion.

## Introduction

Recurrent caries has been the most frequent cause of failure of dental restorations. Fluoride-releasing restorative materials were introduced to overcome this disadvantage. Among the various commercially available fluoride-releasing materials, glass-ionomer cement (GIC) has the highest fluoride release. Resin-modified glass-ionomer cement (RMGIC), compomer, and alkasite, or ion-releasing composite have evolved over the years to harness the advantages of composite resins and GIC. However, the fluoride release of these newer materials is still less than GIC.^1^ Certain inherent properties of GIC, such as anticariogenicity, biocompatibility, adhesion to enamel, dentin, and composite, and its low coefficient of thermal expansion, which is similar to that of tooth structure, make it suitable for a wide variety of clinical applications. Despite these advantages, GIC has certain drawbacks, such as brittleness and porosity, which result in poor mechanical properties, such as low wear resistance and fracture toughness.^2^

The composition of GIC has been experimented with the incorporation of a wide variety of biologically active materials. Modifications of GIC with casein phosphopeptide-amorphous calcium phosphate nanocomplexes (CPP-ACP), calcium sodium phosphosilicate bioactive glass (BAG), and chitosan (CH) have been reported in the literature. The incorporation of CPP-ACP to GIC has shown that the localization of CPP to amorphous calcium phosphate of the tooth surface increases the anticariogenicity by maintaining a state of supersaturation concerning the tooth mineral. Its anticariogenic effect is further potentiated by its interaction with fluoride (F^‒^) ions present in GIC, which produces a stabilized amorphous calcium fluoride phosphate phase.^2,3^

Petri et al showed that the incorporation of CH to conventional GIC (C-GIC) considerably improved its flexural strength and F^‒^ release.^4,5^ The incorporation of BAG into GICs resulted in better dentin mineralization, as it induced calcium phosphate precipitation on its surface in contact with saliva.^6^

Methacryloyloxydodecylpyridinium bromide (MDPB) is a quaternary ammonium compound obtained by substituting the hydroxyl group at the terminal end of hydroxy dodecylpyridinium bromide with the methacryloyl group. This increases the antibacterial activity of the parent compound. MDPB has been a breakthrough in the development of non-agent-releasing antibacterial restoratives.^7^ Imazato et al confirmed the bactericidal activity of MDPB-modified monomer against seven species of oral streptococci.^8^

Despite improvements in the mechanical properties of various modified formulations of GIC, bacterial adhesion on its surface still remains a concern as it predisposes the microenvironment at the tooth‒restoration interface to secondary caries. F^‒^ release from GIC further deteriorates its surface integrity, favoring the adhesion of oral flora.^9^ Several studies have shown that incorporating MDPB into resinous materials like bonding agents and composite resins, resulted in a significant decrease in recurrent caries and bacterial adhesion to the biomaterial surface.^10^ No study has evaluated the effect of incorporating MDPB on the mechanical properties, F^‒^ release, and bacterial adhesion of C-GIC. Hence, this in vitro study aimed to evaluate the effect of incorporating CPP-ACP, BAG, CH, and MDPB on the compressive and flexural strength, F^‒^ release, and bacterial (*Streptococcus mutans* and *Lactobacillus acidophilus*) adhesion of C-GIC.

## Methods

### Preparation of MDPB

The chemicals used to prepare MDPB were of analytical grade. 1-bromododecane and 3-hydroxypyridine were procured from Spectrochem Pvt. Ltd., Mumbai, India. Hydroquinone, acrylic acid, and benzoyl chloride were procured from Merck, Mumbai, India. MDPB was synthesized by the reaction of hydroxy dodecylpyridinium bromide and methacryloyl chloride.

**Figure 1:**
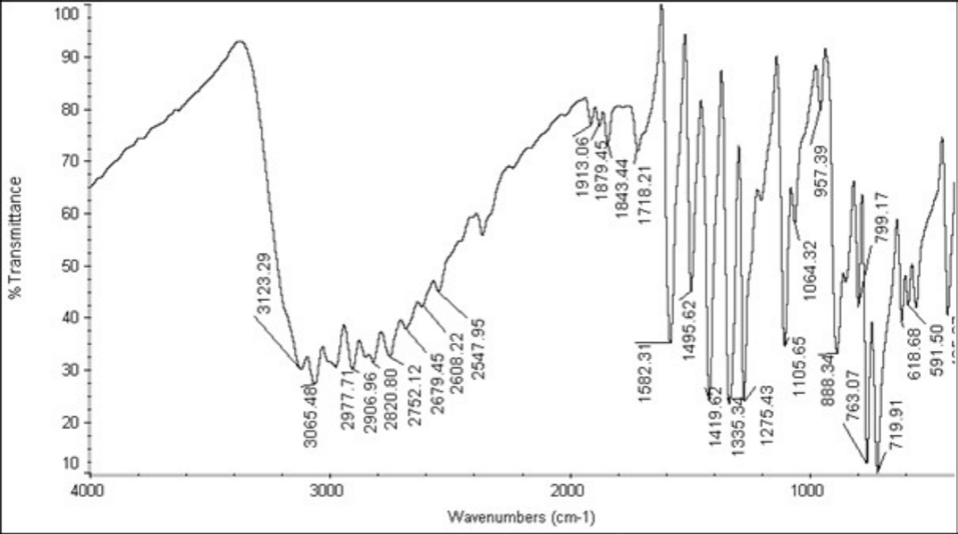
The final product was confirmed as MDPB from the frequencies of infrared spectrophotometry.

0.72 g of acrylic acid was dissolved in 10 mL of distilled ethanol. This solution was added to 1.4 g of benzoyl chloride under 5 mL of 0.1 g of hydroquinone at a temperature of 72‒76°C. The obtained product, acryloyl chloride, was again dissolved in methanol and further subjected to re-distillation at a temperature of 72‒76°C to obtain methacryloyl chloride. Hydroquinone was added as a catalyst to prevent the polymerization of acrylic acid. Hydroxy dodecylpyridinium bromide was prepared from 1-bromododecane and 3-hydroxypyridine by the reflux method. Finally, MDPB was prepared by mixing the prepared hydroxy dodecylpyridinium bromide and methacryloyl chloride at a ratio of 1:1. The obtained product, MDPB, was further confirmed by an infrared (IR) spectrometer. The obtained IR spectrum of the prepared compound was compared with the standard compound, and the frequencies were assigned (Figure 1).

### Preparation of experimental materials

C-GIC (group I) was prepared by dispensing two scoops of powder and two drops of liquid (Type II GIC, GC India Dental Pvt. Ltd., India) on a moisture-impervious paper pad and mixing with an agate spatula in a folding motion for 25‒30 seconds to obtain a paste-like consistency.

Modifications of GIC with CPP-ACP (group II), BAG (group III), and CH (group IV) were implemented by dispensing 180 mg of CPP-ACP paste (Recaldent^TM^, GC Corporation, Japan), BAG powder (45S5, IFGL Bio Ceramics Limited, Kolkata, India), and 36 mg of CH powder (Sigma-Aldrich Co. LLC, USA) to 180 mg of type II GIC powder on separate paper pads and mixing the ingredients thoroughly. To these powders, 120 mg of type II GIC liquid was added and mixed using an agate spatula in a folding motion for 25‒30 seconds to obtain a paste-like consistency. MDPB-GIC (group V) was prepared by first mixing 60 mg of MDPB liquid and type II GIC liquid thoroughly. To this, 360 mg of type II GIC powder was then incorporated and mixed to obtain a paste-like consistency.

### In vitro testing

#### Compressive and flexural strengths

Compressive and flexural strength testing was carried out following ISO specifications 9917-1:2007 and 9917-2:2007, respectively. Stainless steel molds of two dimensions (6 × 4 mm and 10 × 2 × 2 mm) were prepared for compressive strength and flexural strength testing, respectively. Fifteen specimens per group were prepared from the experimental materials for each strength test. During setting, the top and bottom portions of the molds were covered with Mylar strips to obtain a smooth surface. After the initial setting, the test specimens were removed from the molds and stored in artificial saliva for 24 hours. They were then subjected to compressive and flexural loading in a universal testing machine (Instron, Canton, USA) at a 1 mm/min crosshead speed.

### Fluoride release

Under ISO specification 19448:2018, five specimens measuring 6 × 4 mm were prepared using stainless steel molds for each group. After the initial setting, the specimens were stored in 100 mL of deionized distilled water at 37°C. F^‒^ release was evaluated without replenishing the water, at two time intervals of 24 h and seven days with UV spectrophotometry (K. Roy & Co., India) using Erichrome cyanide (Medilab Exports Consortium, Haryana, India) as a reagent.

### Bacterial adhesion tests

For the bacterial adhesion tests, apart from the experimental groups, the polystyrene strip served as a positive control (group VI), and plastic mesh served as a negative control (group VII). Twenty specimens measuring 6 × 4 mm were prepared in each group and randomly divided into two subgroups (n = 10) each, based on the two bacterial strains tested. After the initial setting, the cement specimens were stored in artificial saliva for 24 hours. They were then sterilized in an autoclave at 121°C for 15 minutes at 15-lbs pressure. Under sterile conditions, each test material was placed in the well of a 12-well plate and exposed to a standardized bacterial suspension (2 mL of fresh broth and 20 µL of cell suspension) in brain heart infusion (BHI) broth followed by incubation at 37°C for 4 hours. Reference strains of *S. mutans*and *L. acidophilus*, the common cariogenic oral bacteria, were used. After 4 hours, the test materials were retrieved from the culture broth and washed three times with 5 mL of sterile saline solution to remove non-adhering cells. The test materials were then suspended in glass tubes containing 1 mL of saline solution, and the tubes were transferred to an ultrasonic bath cleaner fitted with a test tube holder, operating at 47 kHz (234 W) and sonicated for 6 minutes to detach the adherent bacteria from the biomaterial surfaces, bringing them into suspension. The test specimens were then removed, and 10 mL of fresh broth was added to each tube. The tubes were again incubated at 37°C for 24 hours. After incubation, the concentration of the bacteria in the broth was finally measured using colorimetry (K. Roy & Co, India). The results were tabulated as optical density (OD) values.

### Statistical analysis

The data were analyzed using one-way ANOVA and Tukey post hoc tests. Significance was set at *P* < 0.05.

## Results

### Compressive and flexural strengths

**Figure 2:**
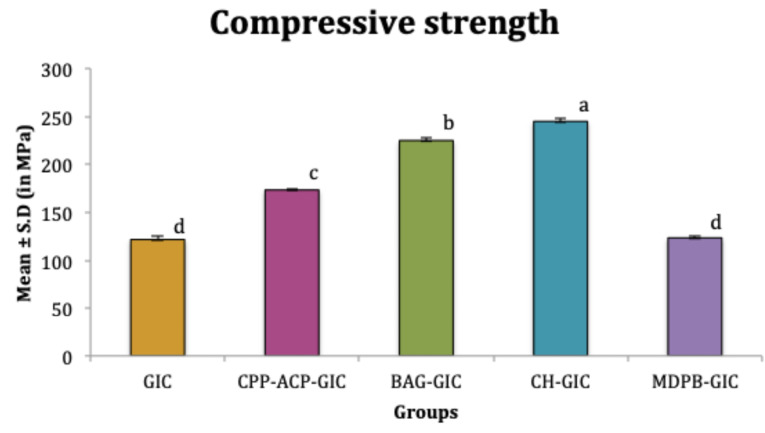
Comparison of mean compressive strengths (MPa) of all the groups. Different alphabets indicate significant difference between the groups (*P*<0.05). SD, standard deviation.

**Figure 3:**
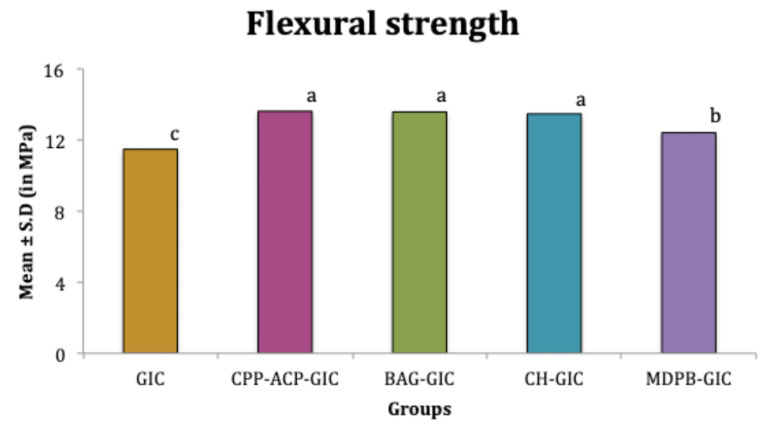
Comparison of mean flexural strengths (MPa) of all the groups. Different alphabets indicate significant difference between the groups (*P*<0.05). SD, standard deviation.

The mean compressive strength of CPP-ACP-GIC, BAG-GIC, and CH-GIC were significantly higher than C-GIC and MDPB-GIC (*P* < 0.05). Among these three, CH-GIC exhibited significantly higher values (*P* < 0.05). No significant difference was observed between the mean compressive strength values of C-GIC and MDPB-GIC (*P* > 0.05) (Figure 2). The mean flexural strength of all the modifications was significantly higher than C-GIC (*P* < 0.05). Among the four modifications, MDPB-GIC exhibited the least flexural strength (*P* < 0.05) (Figure 3).

### Fluoride release

**Figure 4:**
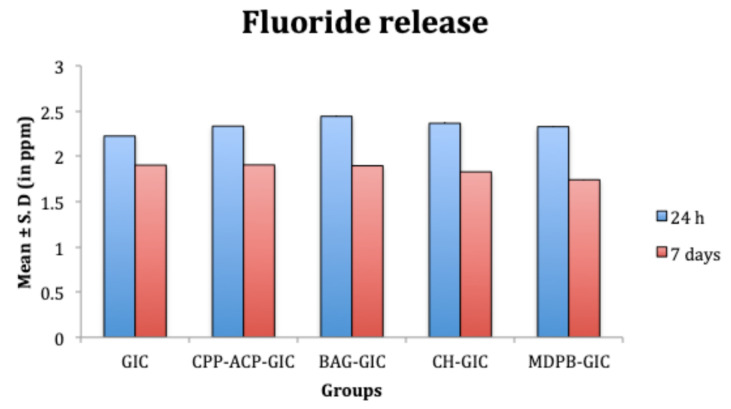
Comparison of mean fluoride release at 24 hours and 7 days.

UV spectrophotometry used in this study to evaluate F^‒^ release offers an advantage over other types as it determines only free ﬂuoride without interference from any type of covalent bond with other ions. At 24 hours, all the modifications showed significantly higher F^‒^ release than C-GIC (*P* < 0.05). Furthermore, BAG-GIC exhibited significantly higher F^‒^release than the other groups (*P* < 0.05) (Figure 4). On day 7, the means of F^‒^ release from C-GIC, CPP-ACP-GIC, and BAG-GIC were significantly higher than CH-GIC and MDPB-GIC (Figure 4).

### Bacterial adhesion

**Figure 5:**
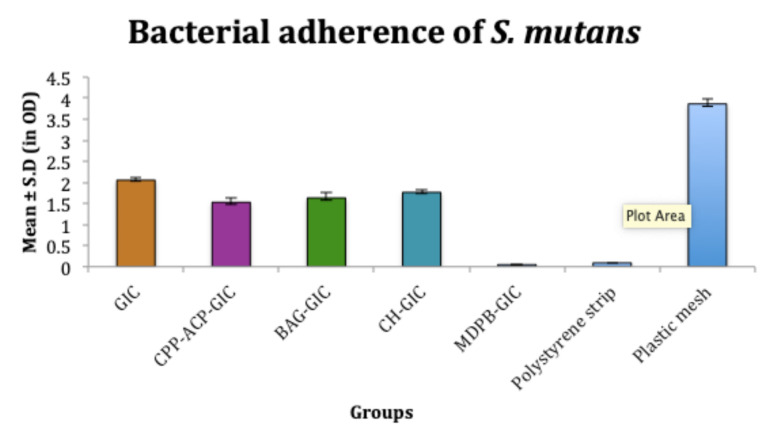
Comparison of mean bacterial adhesion of *Streptococcus mutans.*

**Figure 6:**
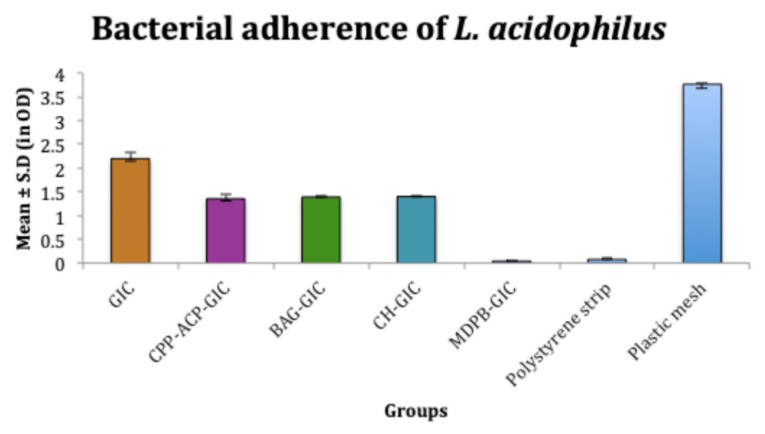
Comparison of mean bacterial adhesion of *Lactobacillus acidophilus.*

According to the results of this study, despite its fluoride-releasing properties, C-GIC showed significantly higher bacterial adhesion (*S. mutans*, 2.05 ± 0.1 OD and *L. acidophilus*, 2.2 ± 0.1 OD) compared to all the experimental groups, but less than the plastic mesh (negative control). The adhesion of *S. mutans* (0.04 ± 0.01 OD) and *L. acidophilus* (0.04 ± 0.008 OD) on MDPB-GIC was significantly less than all the other experimental cements and even less than the polystyrene strip that served as a positive control (0.09 ± 0.01 OD). CPP-ACP-modified GIC exhibited significantly less adhesion of *S. mutans* (1.5 ± 0.01 OD) and *L. acidophilus* (1.3 ± 0.09 OD) than BAG-GIC, CH-GIC, and C-GIC. Adhesion of *S. mutans* (1.5 ± 0.1 OD) and *L. acidophilus* (1.3 ± 0.03 OD) on BAG-GIC was significantly less than C-GIC. CH-GIC showed significantly less adhesion of *S. mutans* (1.7 ± 0.06 OD) and *L. acidophilus* (1.4 ± 0.01 OD) than C-GIC (Figures 5 and 6).

## Discussion

Compressive strength is indirectly related to flexural and diametral tensile strengths in a complicated way. Although direct measurements of tensile strength are inherently valid, problems arise during testing of brittle materials, like GIC. For these reasons, it has been suggested that the measurement of flexural and compressive strengths offers the best practical and reliable estimate of tensile strength.^7^

The increased compressive and flexural strengths of CH-GIC, compared to the other groups, could be attributed to the presence of several hydroxyl and acetamide groups in CH chains, which might bind to the hydroxyl and carboxylic groups of polyacrylic acid (PAA) in C-GIC. This could have reduced the interfacial tension between the glass-ionomer components, improving mechanical performance.^4,11^ The glass particles of BAG adhere to the GIC matrix, reinforcing the matrix by serving as filler components.^12^ C-GIC has a porous structure, with the pores acting as stress concentration points. The incorporation of CPP-ACP nanoparticles leads to the obliteration of these pores, resulting in a cross-linked matrix structure. This could lead to an improvement in the mechanical properties of these modified cements.^2^ The incorporation of an amino acid-derived monomer of MDPB into the PAA of GIC might improve the flexural strength of C-GIC, as shown in a study by Kao et al. It has been reported that the longer the polymer chains of amino acid-derived monomers, the greater is the freedom of movement of carboxylic acid groups to react with the ions leached from the glass particles of GIC. Presumably, this freedom of the primary, secondary, and tertiary pendent carboxylic acid groups improves the reactivity with Ca^2+^ and Al^3+^ ions from the glass, resulting in greater filler‒polymer chemical reaction and increased homogeneity in the cement.^7^

This could be the reason why the carboxylic acid groups present in long polymer chains of MDPB could have enhanced the formation of salt bridges, improving the flexural strength of the modified GIC in the present study, despite a reduction in the volume of PAA by half. However, the mechanism beneath this inference needs to be studied in future experiments.

F^‒^ neither takes part in the acid‒base setting reaction of GIC, nor is it incorporated into the matrix structure.^11^ The highest F^‒^ release in BAG-GIC at 24 hours could be attributed to the higher solubility and dissolution rate of hydroxycarbonate apatite [HCA, Ca_10_(PO_4_)_6_(OH)_2_]_,_ which is formed on the surface of the BAG in an aqueous medium.^12,13^ Similar to BAG, the incorporation of CH might have had a catalytic effect, facilitating the diffusion of F^‒^ through the cement matrix towards the external medium. Additionally, the release of F^‒^ ions from the inorganic matrix seems to be favored when reinforced complexes have been formed.^4^ Reynoldsreported that the release of F^‒^ ions from CPP-ACP-modified GIC was promoted by the formation of CPP-ACP nanocomplexes.^12,14^ The same mechanism could have resulted in an increased F^‒^ release in CPP-ACP-GIC compared to C-GIC at both time intervals in the current study. MDPB-GIC showed no significant difference in its F^‒^ release when compared to C-GIC at both time intervals. Imazatoet al added MDPB to composite resin and reported that MDPB had an immobilized alkylpyridinium part, which was entrapped in the composite resin matrix.^10^ A similar entrapment could have happened within the solid glass matrix of GIC, with no interference with its F^‒^ release.

Despite numerous studies on the morphology of oral biofilm and its effect on the tooth surface, only limited information is available on bacterial adhesion to restorative materials, especially on the surface of F^‒^ releasing restorative materials.^14^ Hence in this study, the adhesion of *S. mutans*and* L. acidophilus*on the different modifications of GIC was evaluated using in vitro adhesion tests. In the present study, the cements were in contact with the bacterial inoculum for 4 hours. This period was selected because complete biofilm formation in the oral cavity usually occurs in 2‒4 hours.^15^

The increased surface roughness of GIC, coupled with the loss of surface integrity associated with fluoride release, predisposes C-GIC to higher bacterial adhesion.^16^ In MDPB-GIC, the strong bactericidal action of MDPB is due to its cationic binding to the bacterial cell wall, which disturbs the membrane integrity, subsequently leading to leakage of cytoplasmic material and bacterial cell lysis.^10^ The polymer chains of MDPB could also have decreased the surface free energy of GIC, resulting in significantly reduced bacterial adhesion.^17^ Reynolds showed that the adsorption of CPP-ACP nanoparticles on the surface of enamel increases the surface net negative charge of enamel, influencing the long-term interactions with microbes through the development of repulsive forces.^12,14^ CPP-ACP has been shown to delay biofilm formation by preventing cell-to-cell adhesion of bacteria. These nanoparticles can also modify the long-term adhesion of streptococci by masking the streptococci-related receptors on salivary molecules.^18^ In BAG-GIC, the surface reaction of BAG with an aqueous medium produces an alkaline solution (pH = 9.8) that can kill the target bacteria. Thus even before a direct contact of bacterial cells with the BAG, they could be killed, reducing cell viability.^19^ CH-GIC exhibited significantly less adhesion. This could be attributed to the cationically charged amino groups of CH, which combine with anionic components, such as N-acetylmuramic acid, sialic acid, and neuraminic acid on the bacterial cell surface, resulting in impaired ion exchange with the medium, chelation of transition metal ions, and enzyme inhibition.^20^ This suppresses bacterial adhesion and growth.

While comparing the F^‒^ release and bacterial adhesion of test materials, the literature shows that little or no correlation exists between the two factors. Hence, despite F^‒^ release, secondary caries ensues beneath and at the margins of the restorations.^21^ Among the experimental groups tested, BAG-GIC, CPP-ACP-GIC, and CH-GIC exhibited higher F^‒^ release, but the adhesion of bacterial strains to these experimental restorative materials was much higher compared to MDPB-GIC, which showed the least F^‒^ release among the experimental groups.

The various parameters in this study were tested under controlled in vitro conditions. Caution should be exercised to extend these results to the clinical settings, where the interplay of multiple factors and complex environmental changes occur. Future studies should assess the biocompatibility, mixing time, setting time, setting reaction, and adhesive properties of these experimental GIC formulations before their effective clinical use. Their durability in stress-bearing areas and individuals with high caries risk also need to be studied.

## Conclusion

The incorporation of CPP-ACP, BAG, and CH improved the compressive and flexural strengths of C-GIC. The incorporation of MDPB did not adversely affect the mechanical properties and F^‒^ release but improved the resistance of C-GIC to bacterial adhesion.

## Authors’ Contributions

Conceptualization: NK and SV. Experimental work: NK. Original draft preparation: NK, SV, VS, SM, and RS. All the authors have read and agreed to the publication version of the manuscript.

## Acknowledgments

The preparation part of MDPB was guided by Dr. B. Karthikeyan PhD., the Department of Chemistry, Chidambaram, Tamil Nadu.

## Funding

This research did not receive any specific grant from funding agencies in the public, commercial, or not-for-profit sectors.

## Competing Interests

The authors deny any actual or potential conflicts of interest related to the study.

## Ethics Approval

Not Applicable.
